# The ‘heat’ goes away: sexual disorders of married women with female genital mutilation/cutting in Kenya

**DOI:** 10.1186/s12978-017-0433-z

**Published:** 2017-12-02

**Authors:** Tammary Esho, Samuel Kimani, Isaac Nyamongo, Violet Kimani, Samuel Muniu, Christine Kigondu, Patrick Ndavi, Jaldesa Guyo

**Affiliations:** 1 African Coordinating Centre for Abandonment of FGM/C, University of Nairobi, Kenyatta National Hospital, P.O Box 19676-00202, Nairobi, Kenya; 2 Department of Community and Public Health, Technical University of Kenya, P.O Box 52426, Nairobi, Kenya; 3 School of Nursing Sciences, Kenyatta National Hospital, P.O Box 19676-00202, Nairobi, Kenya; 4 Cooperative Development, Research & Innovation, The Cooperative University of Kenya, Po Box 24814-00502, Karen, Nairobi, Kenya; 5 School of Public Health, University of Nairobi, Kenyatta National Hospital, P.O Box 19676-00202, Nairobi, Kenya; 6 Thematic Unit of Clinical Chemistry, Department of Human Pathology, University of Nairobi, Kenyatta National Hospital, P.O Box 19676-00202, Nairobi, Kenya; 7 Department of Obstetrics and Gynaecology, University of Nairobi, Kenyatta National Hospital, P.O Box 19676-00202, Nairobi, Kenya

**Keywords:** Female genital mutilation/cutting (FGM/C), Sexual functioning, Female sexual functioning index (FSFI), Cut and uncut women

## Abstract

**Background:**

Female genital mutilation/cutting (FGM/C) has been implicated in sexual complications among women, although there is paucity of research evidence on sexual experiences among married women who have undergone this cultural practice. The aim of this study was to investigate the sexual experiences among married women in Mauche Ward, Nakuru County.

**Methods:**

Quantitative and qualitative data collection methods were used. Quantitative data were obtained from 318 married women selected through multistage sampling. The women were categorized into: cut before marriage, cut after marriage and the uncut. A questionnaire was used to collect demographic information while psychometric data were obtained using a female sexual functioning index (FSFI) tool. The resulting quantitative data were analyzed using SPSS® Version 22. Qualitative data were obtained from five FGDs and two case narratives. The data were organized into themes, analyzed and interpreted. Ethical approval for the study was granted by Kenyatta National Hospital-University of Nairobi Ethics and Research Committee.

**Results:**

The mean age of the respondents was 30.59 ± 7.36 years. The majority (74.2%) had primary education and 76.1% were farmers. Age (*p* = 0.008), number of children (*p* = 0.035) and education (*p* = 0.038) were found to be associated with sexual functioning. The cut women reported lower sexual functioning compared to the uncut. ANOVA results show the reported overall sexual functioning to be significantly (*p* = 0.019) different across the three groups. Women cut after marriage (mean = 22.81 ± 4.87) scored significantly lower (*p* = 0.056) than the uncut (mean = 25.35 ± 3.56). However, in comparison to the cut before marriage there was no significant difference (mean = 23.99 ± 6.63). Among the sexual functioning domains, lubrication (*p* = 0.008), orgasm (p = 0.019) and satisfaction (*p* = 0.042) were significantly different across the three groups. However, desire, arousal and pain were not statistically different.

**Conclusion:**

Generally, cut women had negative sexual experiences and specifically adverse changes in desire, arousal and satisfaction were experienced among cut after marriage. FGM/C mitigating strategies need to routinely provide sexual complications management to safeguard women’s sexual right to pleasure subsequently improving their general well-being.

## Plain English summary

Female genital mutilation/cutting (FGM/C) is all procedures that involve removal of external female genitalia for non-medical reasons. Despite it being implicated with sexual complications among girls and women, there is paucity of research on sexual experiences of married women living with FGM/C. The purpose of this study was to investigate the sexual experiences among three categories of married women; the uncut, cut before marriage, and cut after marriage in Mauche Ward, Nakuru County, Kenya.

A questionnaire was used to collect sexual functioning information from 314 married women. To better understand sexual experiences of married women, focus group discussions and case narratives were also conducted.

Comparison of the three groups revealed lower sexual functioning among the women cut after marriage followed by those cut before marriage, and high among the uncut. Discussions with the married women revealed that married women’s sexual desire changes as they get busy with additional domestic responsibility such as child rearing. When describing the differences in their sexuality before and after being cut, the married women clearly concurred that they encountered challenges in their experiences of sexuality after they got cut. Reasons why women agreed to cut after marriage were explored and they include peer pressure from husbands and mothers-in law, social coercion through stigma and isolation associated to being uncut and to reduce a woman’s sexual desire.

In conclusion, FGM/C negatively affects sexual experiences of married women. There is need to provide routine sexual complications management to women living with FGM/C.

## Background

Female genital mutilation/cutting (FGM/C) is a deep rooted cultural practice associated with various medical, psychological and sexual health complications, and is a violation of the social and human rights of victims [1, 2]. It involves the partial or total removal of the external genitalia or other injuries thereof for cultural or non-medical reasons [3, 4]. Four types of FGM/C namely clitoridectomy (Type I), excision (Type II), infibulations (Type III) and all other harmful procedures such as labia pulling, piercing and cauterization (Type IV) are documented [5].The current global magnitude of FGM/C stands at 200 million girls and women who have been forcibly cut. Moreover, three million girls are at risk each year, translating to 30 million girls and women vulnerable to any form of cutting in the next decade [6, 7].

Female genital mutilation is practiced globally by ethnic groups found mainly in 30 African countries (East, North East, and West Africa), Middle East, Asia, Latin America and Western nations among immigrant populations [5, 8–10]. Among immigrants, the practice is mainly conducted during return home (visiting/holiday) trips [11, 12] but significant female cutting is also performed in the Western (Europe, North America and Australia) countries as a way of perpetuating the carried along culture [12–15]. In Kenya, FGM/C is widely practiced by majority of communities, with exception of the Luo, Luhya, Pokomo, Teso and Turkana out of the 42 ethnic groups. FGM/C among 15–49 year olds has steadily decreased from 37.6% (1998) to 32.2% (2003) then 27.1% (2008–9) and currently 21% [16].

Although FGM/C has been implicated in sexual complications among girls and women, there is paucity of research on the sexual experiences of married women with FGM/C (or simply, cut women). Evidence point to the fact that cut women were more likely to report painful intercourse, reduced sexual desire, less sexual satisfaction and less experience of orgasm compared to their uncut counterparts [17]. Other sexual outcomes reported include women not initiating sex and lack of knowledge of the most sexually sensitive part of their bodies [18]. Among the Kipsigis who live in Mauche location, Nakuru County in Kenya, girls are cut in their adolescence but a new trend of women undergoing the cut at mature age is emerging (long after getting married and having children). This study investigated the sexual experiences among three categories of married women - uncut, cut before marriage, and cut after marriage - in Mauche location, Nakuru County.

## Methods

**Fig. 1:**
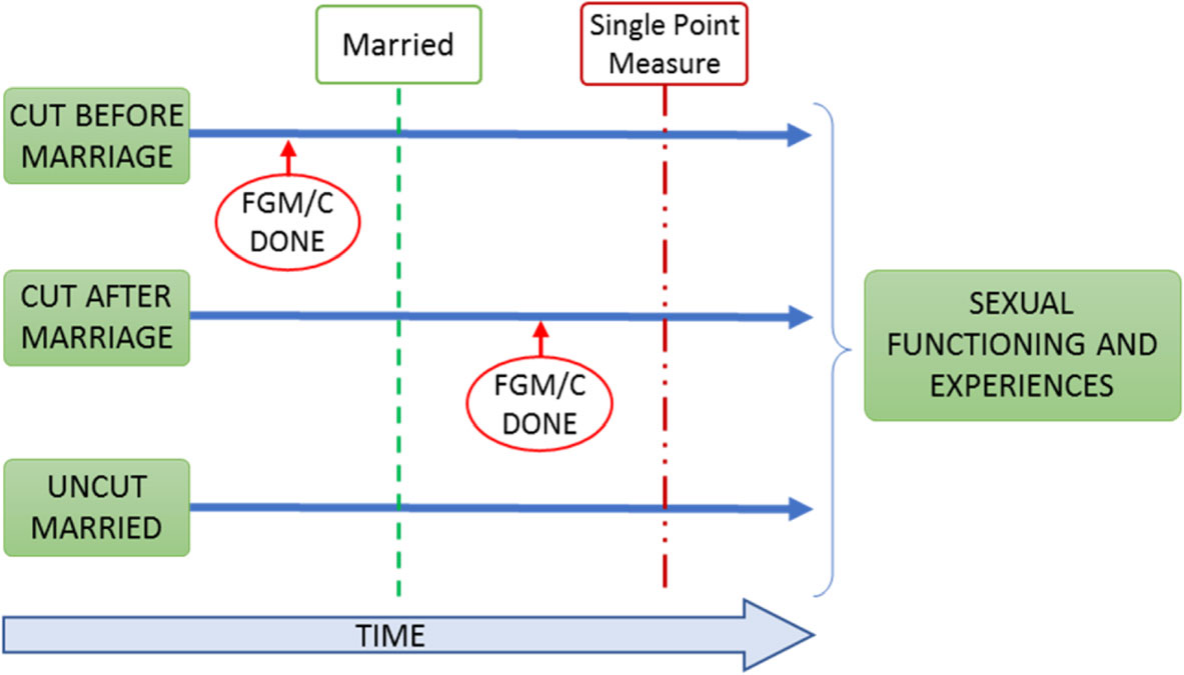
Study design

The study used mixed methods where both quantitative and qualitative data were collected between November 2015 and March 2016. The quantitative component adopted a comparative cross sectional design as illustrated in Fig. 1. The 318 married women in this study were identified from a larger study of 377 women who participated in the study comparing sexual functioning between the cut and the uncut women. The 318 were categorized into three groups thus: cut before marriage (*n* = 140), cut after marriage (*n* = 29) and uncut (*n* = 145) were identified. Four respondents were excluded from this study because, although married and cut, it was not indicated when they were cut.

The quantitative data were collected using the demographic questionnaire and psychometric instrument notably the female sexual functioning index (FSFI) [19–21]. The demographic questionnaire captured age, level of education, number of children, economic activity and cutting status. The psychometric instrument is a validated tool with 19 questions capturing six female sexual functioning domains namely, sexual desire, arousal, lubrication, orgasm, satisfaction, and pain. The data collection tools were translated to local language (Kipsigis) and back-translated to English. The demographic questionnaire and the psychometric instrument were interviewer administered using well-trained research assistant.

The quantitative data were cleaned, entered and analyzed using Statistical Package for Social Sciences (SPSS®) version 22. For the psychometric instrument, each question was given a score ranging from 0 to 5. A domain score was calculated by multiplying the sum scores of each question in each domain by a pre-determined domain factor [19]. The domain factors were as follows: desire had 0.6; arousal and lubrication had 0.3, while orgasm, satisfaction and pain had 0.4, respectively. The full-scale scores for each Respondent were calculated by adding all the domain scores. The minimum score a Respondent could get was 2 and the maximum was 36. To summarize the quantitative data, the numerical variables were analyzed into means and standard deviations while the categorical variables into frequency Tables. A one-way ANOVA and independent t-test were used for bivariate analysis. Multivariate linear regression analysis was performed with FSFI scores as dependent variable and the socio-demographic characteristics that were significant (*p* < 0.05) at bivariate analysis and the cutting status (cut/uncut) of the women as independent variables. A *p*-value of less than 0.05 was considered significant.

The qualitative method collected data using discussion and case narrative guides based on the 6 FSFI domains as the themes. Five focus group discussions and two case narratives were conducted. The discussions were audio and notes manually recorded. Transcriptions were conducted and verbatim statements for each theme were selected. The verbatim statements supplemented the quantitative findings. Ethical approval for the study was granted by Kenyatta National Hospital-University of Nairobi Ethics and Research Committee (KNH/UON ERC) (Approval Number P96/02/2015). The study participants gave both verbal and written consent.

## Results

### Socio-demographic characteristics of respondents

The Respondents were 314 married women distributed as follows: cut before marriage 140 (44.6%), cut after marriage 29(9.2%) and uncut 145 (46.2%). The mean age of the Respondents was 30.59 ± 7.36 years ranging from 15 to 45 years. About three-quarters (76%) of the Respondents were 25 years and above. An analysis with chi-square test of independence was performed to examine the relation between social demographic factors and the cut status of the married women. Respondents who were uncut were more likely to be young (χ^2^ = 34.885, df = 2, *p* < 0.001), have fewer children (χ^2^ = 45.885, df = 2, p < 0.001), and of higher education (χ^2^ = 20.888, df = 2, p < 0.001) (Table 1). With regard to economic activity, over three-quarters (76.1%, *n* = 246) were farmers. Chi-square test of independence found no relationship between economic activity and the cutting status of the married women.

**Table 1:** Socio-demographic characteristics in relation to cut status

Characteristics	Cut after marriage (%)	Cut before marriage (%)	Married & Uncut (%)	Total (%)	χ^2^	df	*p*-value
Age					34.885	2	<0.001
15–24 years	10 (13.0)	12 (15.6)	55 (71.4)	77 (100.0)			
25+ years	19 (8.0)	128 (54.0)	90 (38.0)	237 (100.0)			
Number of children					45.983	2	<0.001
0–3 children	11 (8.8)	28 (22.4)	86 (68.8)	125 (100.0)			
4+ children	18 (9.5)	112 (59.3)	59 (31.2)	189 (100.0)			
Highest level of education					20.888	2	<0.001
0 to Primary	25 (10.2)	124 (50.4)	97 (39.4)	246 (100.0)			
Secondary +	4 (5.9)	16 (23.5)	48 (70.6)	68 (100.0)			
Economic activity					0.180	2	0.914
Farmers	23 (9.6)	106 (44.4)	110 (46.0)	239 (100.0)			
Others	6 (8.0)	34 (45.3)	35 (46.7)	75 (100.0)			
Total	29 (9.2)	140 (44.6)	145 (46.2)	314 (100.0)			

#### Sexual functioning

An independent t-test reveals a link between sexual functioning and three socio-demographic characteristics namely age, number of children and education. The data shows a significant association (*p* = 0.008) between age and sexual function with the younger women showing higher FSFI scores compared to older women (25.25 vs 23.67). In addition, there was a significant association (*p* = 0.035) between number of children and sexual functioning. The women with fewer children had higher FSFI scores compared to women with more children (24.84 vs 23.42). Similarly, there was a significant association (*p* = 0.038) between educational level and sexual functioning with post primary educated women having higher FSFI scores compared to those with primary education (25.64 vs 24.15). These findings are corroborated by data from FGDs and case narratives.

Women reported that children may lead to reduced sexual desire among married women. Indeed, there was concurrence that married women’s sexual desire changes as they get busy with additional domestic responsibility such as child rearing. *“I think it's when the love is now going down, by this time there are children responsibilities, work, and life is generally harder”.* (Young Women, FGD,, Mauche Shopping Centre).

Women’s sexual experiences are captured vividly in various case narratives. The excerpt below, from a middle-aged woman (35 yrs) and mother of 4 who was forced to cut 6 months after giving birth to her first baby, is a typical representation of experiences of cut women. It reveals bitterness and frustration in her sex life;

*There was a lot of change because the body does not feel excited for sex even if you see a man you just see that you are not interested at all. Before I was cut, I used to have a lot of desire for sex such that we would even stop our meal halfway to go and I would tell my husband to go and have sex first. My husband told me that he used to enjoy sex more before I got cut than now when I am cut….I don’t feel like having sex at all, the body doesn’t want it and the mind is full of other things that are stressing me…. If it had been today I would refuse to get cut. Had I known better….* (35 years old woman, Case Narrative 1, Mauche Shopping Center)

Married cut women in this community clearly concurred that they encountered challenges in their experiences of sexuality after they got cut. They described the differences in their sexuality before and after being cut. The following narrative points to some of these sexual challenges that cut women have to contend with. This is reiterated by a participant in one FGD. “*The ‘heat’ goes away [after being cut] so the wife is just forced by her husband [to have sex] when she is sleeping”* (Participant 5, Cut participant, FGD with cut and uncut women, Mauche Shopping Center). Another FGD participant reiterated, “*When you have been cut, your husband remains ‘hot’ and then he forces you to have sex [and] because you just don’t feel like, it [sex] is not sweet….”* (Participant 1, FGD with cut and uncut women, Mauche Shopping Center).

In order to measure these experiences objectively, the sexual functioning of cut and uncut women in Mauche was assessed across the six domains of the FSFI tool namely: desire, arousal, lubrication, orgasm, satisfaction and pain. Overall the married uncut women had better sexual functioning scores (25.35) compared to cut before marriage (23.99) and cut after marriage (22.81), and the differences were statistically significant (*p* = 0.019). Further analysis with Bonferroni post hoc test revealed that women cut after marriage scored significantly (*p* = 0.056) lower than the uncut. However, in comparison to the cut before marriage there was no significant difference. Lubrication, orgasm and satisfaction were found to be statistically different across the three groups while desire, arousal and pain (reverse-scored) showed no statistical difference (Table 2). Lubrication scored significantly (*p* = 0.013) lower among women cut before marriage than the uncut. However, in comparison to cut after marriage there was no significant difference. Both orgasm (*p* = 0.041) and satisfaction (*p* = 0.043) scored significantly lower among women cut after marriage than the uncut and no significant difference with those cut before marriage.

**Table 2:** Women sexual functioning scores in relation to cut status

	Cut after marriage		Cut before marriage		Married & uncut		*P*-value
	(*n* = 29)		(*n* = 173)		(*n* = 145)		
*Domain*	Mean (Min - Max)	SD	Mean (Min - Max)	SD	Mean (Min - Max)	SD	
Desire	3.35 (1.20–5.40)	1.14	3.38 (1.20–6.00)	1.31	3.55 (1.20–6.00)	1.09	0.402
Arousal	3.70 (2.10–5.40)	0.95	3.82 (0.00–6.00)	1.47	3.96 (0.00–6.00)	0.93	0.472
Lubrication	3.66 (1.80–5.40)	0.97	3.76 (0.00–6.00)	1.4	4.14 (2.10–6.00)	0.86	0.008
Orgasm	3.82 (1.60–6.00)	1.3	4.15 (0.00–6.00)	1.47	4.45 (2.00–6.00)	0.96	0.019
Satisfaction	4.21 (2.40–6.00)	1.09	4.75 (0.40–6.00)	1.21	4.78 (1.60–6.00)	1.07	0.042
Pain	4.7 (2.40–6.00)	1.41	4.18 (0.00–6.00)	1.59	4.47 (1.20–6.00)	1.16	0.142
Score	22.81 (13.30–28.90)	4.87	23.99 (3.60–35.60)	6.63	25.35 (17.90–33.60)	3.56	0.019

A multivariate linear regression analysis was performed with female sexual function index scores as dependent and cutting status, age, number of children and level of education as predictor variables (Table 3). Tolerance values for the four predicator values were between 0.518 and 0.915 indicating there was no multicollinearity. The analysis revealed a significant model, accounting for 6.6% of the variance in sexual function, *F*(4, 309) = 5.437, *p* < 0.001, *R* ^*2*^ = 0.66. The analysis showed that age (β = −0.181, *p* = 0.018) and highest level of education (β = 0.162, *p* = 0.005) were independent predictors of FSFI total scores. The scores decreased by 0.13 for 1 year increase in age and increased by 1.577 for increase in level of education.

**Table 3:** Multivariate linear regression analysis for cutting status and socio-demographic characteristic in relation to female sexual function index scores

Variable	B	SE	β	t	p	Collinearity	
						Tolerance	VIF
Constant	25.195	2.053		12.273	0.000		
Cutting status (Cut or uncut)	−0.590	0.671	−0.056	−0.880	0.380	0.759	1.317
Highest level of education	1.577	0.558	0.162	2.826	0.005	0.915	1.092
Age	−0.130	0.055	−0.181	−2.369	0.018	0.518	1.930
Number of children	0.165	0.179	0.070	0.923	0.357	0.519	1.927

Reasons why women agreed to cut after marriage were explored and they include peer pressure on the husband and stigma associated to being different. One participant observed that *“friends of the husband put a lot of pressure by refusing to eat her [his wife’s] food until she is cut”* (Older women 42–50 years, FGD, Chief’s camp). Another added, “*because the woman is called ‘ugali mbichi’ (partially cooked maize meal), she is seen as a child …”.* Other reasons given for husbands forcing their wives to be cut include an attempt to reduce the women’s perceived ‘high’ sexual desire. This is captured in the following quote from a participant in one FGD, *“some husbands feel like wives have too much ‘heat’, hence they want to reduce* [it]” *(*Young Women,FGD, Mauche Shopping Centre). Stigma against women who are not cut was expressed as, *“the state of being despised will be so much, you cannot be a community leader”* (Participant 4), with another adding her view on how to deal with this stigma, *“it is better you just accept to be cut”* (Participant 3).

The socially moderated sexual behavior subdued women’s overt expressions of their sexuality as captured below:

*A married woman is not expected to initiate sex even when she may desire lest this is misinterpreted for immorality*…. (Participant 2, FGD of older women, Chief’s camp Mauche)

*… you cannot tell your husband that you want sex, so you wait for him to ask. If you ask he will think you are ‘lustful’ and can be mistaken for being a prostitute*. (Participant 1, FGD of older women Chief’s camp Mauche)

While the expected proper behavior may be culturally moderated, and even negatively affected by FGM/C, such moderation is not absolute as revealed in a view expressed by one of the participants from the older women’s FGD. This woman, although cut, mentions that she sometimes takes initiative to sexually arouse her husband although she associates it to trying to please him when he is stressed out. She stated; *“When you are married you get to know your husband and when he is unhappy you can begin touching him in bed but you will not talk about it (sex)* (Older women, FGD, Chief’s camp Mauche). This shows that as much as sexual experiences may be altered negatively by FGM/C, women reportedly experience sexual desire and enjoyment.

## Discussion

This study investigates sexual experiences of married women in the Mauche community. The findings indicate that FGM/C adversely affects their sexual experiences. The results are discussed along two dimensions: demographic differences between the cut and uncut women, and sexual functioning between uncut and cut women. We also discuss our experience using the FSFI tool.

### Demographics between cut and uncut women in relation to sexual functioning

Several studies have shown a link between socio-demographic factors and sexual functioning [19, 22, 23]. In this study demographic characteristics had an impact on women’s sexual functioning. The majority of the cut women were elderly and had more children compared to the uncut. There is a correlation between women who were cut, were older, had more children and lower levels of education on one hand and low sexual functioning scores on the other. This finding concurs with a study conducted in Egypt investigating sexual functioning of women with FGM/C and concluded that demographic characteristics were strongly predictive of sexual difficulties [24]. Concerning age, older women in our study sample reported more sexual problems compared with the younger ones. It is this same group of women that also had more children. This could be confounded by the fact that these women also had lower levels of education and therefore were not very much exposed to the modern way of life, with access to informative or educational materials, access to technology among other things thus affecting their understanding about sexual and reproductive health. The data also suggests that the increase in sexual difficulties among this group was associated with the increasing demands of growing children, running an expanding family and the stresses of a physically demanding lifestyle. Various other studies using the FSFI tool have shown that age and lower levels of education are predictors of female sexual dysfunction [25–27]. However, although having similar conclusions, the Taiwanese [26] and Chinese [27] studies were about pregnant and all women respectively.

### Sexual functioning between uncut and cut married women

The reported overall sexual functioning among women who had undergone FGM/C was lower compared to the uncut ones. Specifically based on the FSFI tool, lubrication, orgasm and satisfaction were reported to be lower in cut women relative to the uncut. However, other domains notably desire, arousal and pain were not statistically different. This portends that FGM/C and its effects thereof adversely affect sexual functioning. These findings are consistent with reports among Arabian women who had undergone FGM/C had lower scores for arousal, lubrication, orgasm, and satisfaction as well as the overall sexual functioning scores [17]. The findings can be explained biologically because clitoridectomy may affect the pleasure point and cutting of female external genitalia arguably adversely affect sexual tissue and sensations affecting the experiences among cut women [22, 28, 29]. Another study also correlated FGM/C and reduced sexual quality of life based on the results of the Sexual Quality of Life-Female questionnaire through a study conducted in England [30]. It is thus crucial to realize that, a reduced overall sexual functioning may impact on the women’s sexual quality of life and subsequently of their overall general well-being.

On the other hand, the finding that desire and arousal were not significantly different between cut and uncut contrasts a study that investigated pleasure and orgasm among women who had undergone FGM/C and which found that cut women including infibulated ones had higher orgasm and that desire, arousal, orgasm, and satisfaction were higher among the infibulated than the uncut women, although lubrication and pain were not statistically different between the two groups [31]. These findings could be explained by the fact that some erectile structures fundamental for orgasm have not been excised during FGM/C [31]. However, the difference in findings could also have been due to methodological differences, sample and location of the study. More recently, a well-controlled cross-sectional study on sexual functioning in women with and without genital mutilation, found no difference between orgasm, desire and satisfaction [32], a variance that could have been due to sample size.

There was a significant difference in lubrication, orgasm and satisfaction among the three study groups. Women who were cut had problems with lubrication during intercourse, which could be explained by the fact that they also have lower sexual desire and no orgasm. This could also be compounded by the lack of sexual enjoyment due to other factors such as sexual pain experiences. Damage to the female external genitalia specifically damage to the bartholins and vestibular glands could also result in this finding.

Women exposed to FGM/C after marriage scored lower than the uncut. Specifically, there was a significant difference in satisfaction between them and those cut before marriage. We suspect that these women had experience about their sexual satisfaction before they got cut and thus have an idea of the difference compared to those cut before marriage who cannot distinguish the two experiences. The difference on the principal component of satisfaction is that FSFI measures satisfaction with amount of closeness with partner, with sexual relationship and with overall sex life which are a combination of more external-internal factors as compared to the other domains which are more organic in nature and thus explains the difference between sexual functioning of cut before and after marriage. Sexual satisfaction has been defined as “an affective response arising from one’s subjective evaluation of the positive and negative dimensions associated with one’s sexual relationship” [33]. Due to satisfaction being an affective response, it means that women with FGM/C have a possibility of experiencing sexual satisfaction if they have fulfilling interpersonal relationships with their spouses despite their cutting status.

### Our experience using the FSFI tool

The FSFI tool has been used to measure various domains - desire, arousal, lubrication, orgasm, pain and satisfaction - of female sexual functioning under different contexts. This study set out to measure the sexual functioning of married women who had undergone the cut (before and after marriage) and those that had not. Based on the results of the factor analysis of the 19-item questionnaire, there were similar factor loadings for desire, arousal, lubrication, orgasm and pain between cut before and after marriage. However, there is a significant difference in satisfaction between cut before and after marriage. This difference on the principal component of satisfaction perhaps results from the fact that the FSFI measures satisfaction with amount of closeness with partner, with sexual relationship and with overall sex life. It is, thus, a combination of more external-internal factors compared to the other domains which are more organic in nature and thus explains the difference between sexual functioning of cut before and after marriage. Consequently, within our context the tool is more sensitive to the satisfaction domain compared to the rest of the domains covered by the tool.

Although the tool is developed and validated in a different cultural context, we are confident that it was able to capture and measure the various domains within the rural Mauche community. It is, however, clear that our measures are consistently lower than the recommended cut-off point of 26 [19]. We ask the question: why these differences? Across various cultural groups, there are different expectations regarding sexual relations and particularly in terms of how women and men express their sexuality. Overall, women across all cultures tend to suppress their sexual expression compared to men who tend to exaggerate those issues [1]. Indeed, in many African communities, an expression of sexual desire, for instance, by a woman could easily be construed as an outward admission of a promiscuous individual, an undesired label. Thus, from an early age women tend to suppress any expressions that might portray this negative image. To what extent the FSFI tool is sensitive to issues like sexual desire is a matter of conjecture. As we point out here, the scores using the FSFI tool are consistently lower than the cut-off point but they are consistent with our expectation in terms of the three groups namely women who got cut after marriage would have lower scores compared to those who got cut before marriage while those who have not been cut would have the highest scores. Furthermore, in the translation of the tool from English to the local language, it was not always possible to get an exact equivalent word. This could result in loss of sensitivity of the tool. Nonetheless, efforts to translate the tool in other contexts have resulted in a tool that closely measures sexual functionality of literate women with a sensitivity similar to the English tool (see for example [34]). We feel that a tool that responds better to these cultural differences or one that includes a correction factor would help standardize the findings from different cultural contexts and backgrounds.

#### Further research and dissemination

This study has revealed married women’s sexual functioning and sexual experiences from Mauche. A comparative study to measure men’s sexual experiences with either cut and/or uncut women would add men’s voice to either corroborate the women’s views or provide insight into men’s views regarding how FGM/C influences sexual experiences or functioning. Further, a comparison of experiences based on different types of FGM/C would help distinguish sexual experiences along the sliding scale of the cut type. Moreover, an investigation of the overall sexual quality of life of the women may reveal the general impact of FGM/C on women’s sexual quality of life.

## Conclusion

This study has revealed that sexual experiences and functioning of married women among the Kipsigis, a sub-group of the Kalenjin community, of Mauche, are negatively affected by FGM/C. The sexual experiences of the women could be subjective but this has also been substantiated with the measurements using the objective tool FSFI which further revealed the impact of FGM/C on their sexual functioning. This negative impact is strongest on lubrication, orgasm and satisfaction and less so on desire, arousal and pain. The effect of a reduced sexual functioning may lead to poor sexual quality of life which is likely to negatively impact the women’s general well-being. Therefore, FGM/C mitigating strategies should incorporate sexual complications management to safeguard women’s sexual right to pleasure, subsequently improving their general well-being. Furthermore, coordinated efforts through multifaceted strategies must be sustained and escalated for the abandonment and mitigation of FGM/C.

## Abbreviations

ANOVA: Analysis of Variance
FGD: Focus Group Discussions
FGM/C: Female Genital Mutilation/Cutting
FSFI: Female Sexual Functioning Index - a validated psychometric instrument for measuring female sexual functioning
KNH/UON ERC: Kenyatta National Hospital - University of Nairobi Ethics and Research Committee
SPSS: Statistical Package for Social Sciences
